# The degree and source of plastic recyclates contamination with polycyclic aromatic hydrocarbons

**DOI:** 10.1039/d0ra08554e

**Published:** 2020-12-21

**Authors:** Ayah Alassali, Wolfgang Calmano, Evangelos Gidarakos, Kerstin Kuchta

**Affiliations:** Hamburg University of Technology – Institute for Environmental Engineering and Energy Economics – Sustainable Resource and Waste Management Blohmstrasse 15 D-21073 Germany ayah.alassali@tuhh.de; Technical University of Crete, School of Environmental Engineering Polytechneioupolis 73100 Chania Greece

## Abstract

In this research, the degree and source of recyclates contamination with polycyclic aromatic hydrocarbons (PAH) was studied in eight different polyolefin recyclate samples; four originating from post-consumer packaging waste and four originating from a mixed source (post-industrial, post-commercial, and post-consumer). The aim was to assess the applicability of these recyclates in the different products' categories. Furthermore, the impact of previous contamination with PAH was excluded by analysing pure plastics before and after undergoing simulated recycling processes. Polythene recyclates originating from post-consumer plastic packaging waste had lower concentrations of the 16-US-EPA PAH (922.15 ± 420.75 μg kg^−1^) in comparison to the ones of a mixed origin (2155.43 ± 991.85 μg kg^−1^), *r* = −0.35, *p* > 0.05. The degree of recyclates contamination with PAH was always within the REACH limits for consumer products (<1.0 mg kg^−1^). On the other hand, only polythene recyclate sample originating from post-commercial waste did not comply with the REACH limits for children articles (0.5 mg kg^−1^). Hence, the source of plastic waste defines the quality of recyclates. All in all, the results indicated that the contamination of polyolefin recyclates with PAH is attributed to the material's previous contamination, or the sorption of plastics to organic compounds from the surrounding environment. Exposing plastics containing PAH additives to heat during extrusion could result in further accumulation of PAH in plastics.

## Abstract 2

The degree and source of recyclates contamination with polycyclic aromatic hydrocarbons was studied in 8 polyolefin recyclate samples; 4 from post-consumer packaging waste and 4 from a mixed source (post-industrial, post-commercial, and post-consumer).

## Graphical abstract

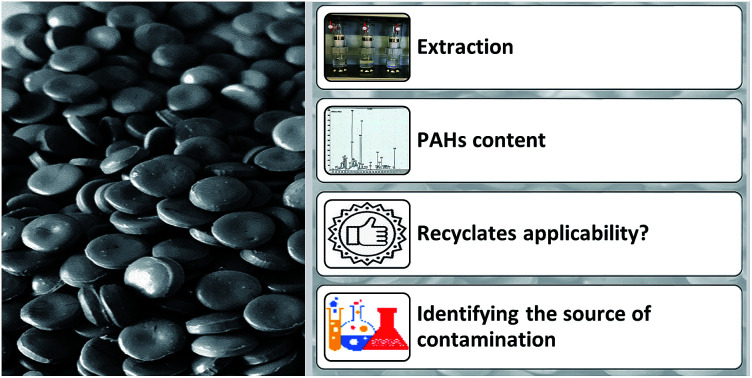

## Introduction

The plastic waste problem is becoming a global priority, hence, provoking stakeholders to take actions enhancing the existing waste management systems and to invest in new ones. For instance, the plastic industry is striving to boost the impact of a circular economy on plastics,^1^ where resources are maintained and the maximum value is recovered after disposal.^2^

The plastic mechanical recycling process includes different phases of material processing. They comprise sorting, shredding, washing, melting and/or regranulating steps that could be joined in different schemes.^3–5^ Generally, the practices involved in the mechanical recycling process expose plastics to heat, physical stress, and oxidation, which will eventually affect the quality of recyclates.^3,6–8^

Due to the need for high quality recyclates from such a heterogeneous waste stream, shortcomings of the mechanically recycled plastic should be addressed, particularly with including the up-to-date regulations on substances, mixtures and products. The presence of unwanted chemicals that can migrate and accumulate in the plastic's cycle is a significant limitation and risk to its closed-loop recycling.^9–12^

Polycyclic aromatic hydrocarbons (PAH) are among the contaminants of concern. They have toxic, carcinogenic and mutagenic characteristics.^13,14^ Many of the PAH substances are categorised as persistent, bio-accumulative and toxic (PBT) substances. These substances do not degrade and can remain for extended periods in the environment.^13^ Polycyclic aromatic hydrocarbons can be found in plastics, primarily as a result of additives added during the production and manufacturing stages. There are two main additives considered as a source of PAH in plastics, *i.e.* carbon black and extender oils.^15,16^ Products on the market for supply to the public and containing polycyclic aromatic hydrocarbons (PAH) were restricted by entry 50 of Annex XVII of the Regulation (EC) No 1907/2006 of the European Parliament and of the Council of 18 December 2006 concerning the Registration, Evaluation, Authorisation and Restriction of Chemicals (REACH),^17^ paragraphs 5 and 6.^18,19^ The restriction applies on products put on the market after 27 December 2015 and containing one or more of the eight priority PAH defined by REACH. The defined threshold limits are: 0.5 mg kg^−1^ for children articles and 1.0 mg kg^−1^ for all other consumer products.^20^

In a study done by Camacho *et al.*,^21^ aromatic hydrocarbons concentrations were five times higher in recycled resins in comparison to virgin plastics. Generally, it was reported that the number of chemicals present in recycled plastics is higher in comparison to new plastics. Low molecular substances, such as alcohols, esters, ketones and fragrance, and flavour compounds were absent in pristine resins, yet detectable in recyclates. The literature also discussed the emissions of contaminants (including polycyclic aromatic hydrocarbons (PAH), volatile organic compounds (VOCs), phthalate esters, and heavy metals) by the recycling process.^22–24^ Nonetheless, it was not anticipated that these contaminants are accumulated in the recycled material.

Contamination with PAH has been studied in plastic and rubber products as a result of the added carbon black or extender oils.^25^ Yet, plastic contamination by PAH due to the stress posed by the mechanical recycling process was not previously discussed.

This research had two main objectives. Firstly, to quantify the degree of polyolefin recyclates contamination with PAH to define the limitations in their applications. Secondly, to evaluate the impact of the mechanical recycling process on plastics contamination with PAH by applying lab-simulated mechanical recycling on pristine polythene (PE) and polypropylene (PP) samples. The aim was to control the number of variables affecting the PAH content in recyclates. The samples were tested before and after applying 3 cycles of extrusion. Hence, it was possible to assess the potential of PAH accumulation in polyolefin due to its mechanical recycling.

The outcomes of this research can help in understanding if the mechanical recycling process of plastics could be resulting in quality deterioration due to the organic contamination with polycyclic aromatic hydrocarbons. Consequently, recommendations to the recycling process could be concluded.

## Materials and methods

### Virgin and pure plastics

In the context of this research, plastics extrusion was applied to simulate the effect of the mechanical recycling on pristine (new and pure) low-density polythene (LDPE) granules (3 mm diameter × 3 mm height) as well as polypropylene (PP) pellets (∼3 mm diameter).

The LDPE granules and PP pellets were purchased from INEOS Olefins and Polymers Europe.

The extrusion of plastics was performed using ZSE 27 maxx (a twin-screw extruder from Leistritz), with a screw torque of 134 Nm per screw, total power of 46 kW and 12 heating zones (including the die). The applied rotational speed ranged between 100 and 150 rpm with an extrusion speed of 3 kg h^−1^. The first extrusion cycle was applied on 5 kg of virgin granules. After each extrusion cycle, 1 kg of material was separated for testing purposes and the rest was dried overnight in BINDER ovens at 60 °C before being sent for shredding and further extrusion. This was repeated until the three extrusion cycles for each polymer were accomplished.

### Recycled polymers

#### Recyclates purchased from recyclers without further processing

The quality of recycled plastics (available on the market) in terms of contamination with polycyclic aromatic hydrocarbons was assessed. The aim was to test their application viability in consumer products and children articles. For that reason, six different HDPE recyclate samples (PE1, PE2, PE3, PE4, PE5, and PE6) and two different PP recyclate samples (PP1 and PP2) were purchased (see Table 1 for details).

**Table 1:** Polyolefin recyclate samples purchased from the market for PAH analysis

Sample name	Image	Description	Source material
PE1	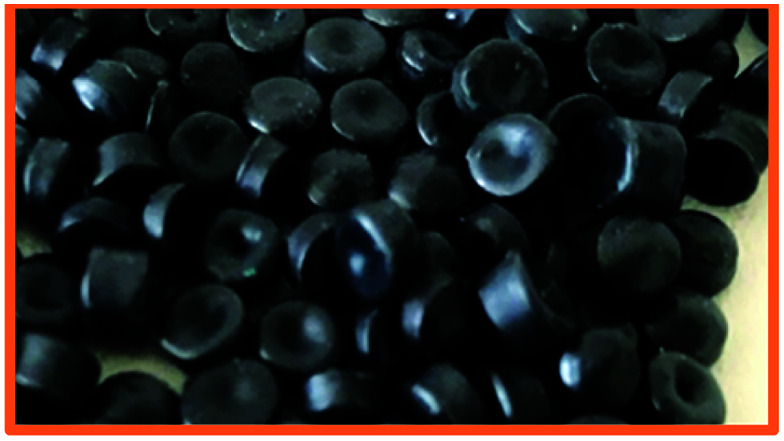	Anthracite cylindrical pellets	Pre-sorted plastic blends of mixed origin (post-industrial, post-commercial, bulky waste, and post-consumer). A selective recycling technology is applied, including degassing and filtration
PE2	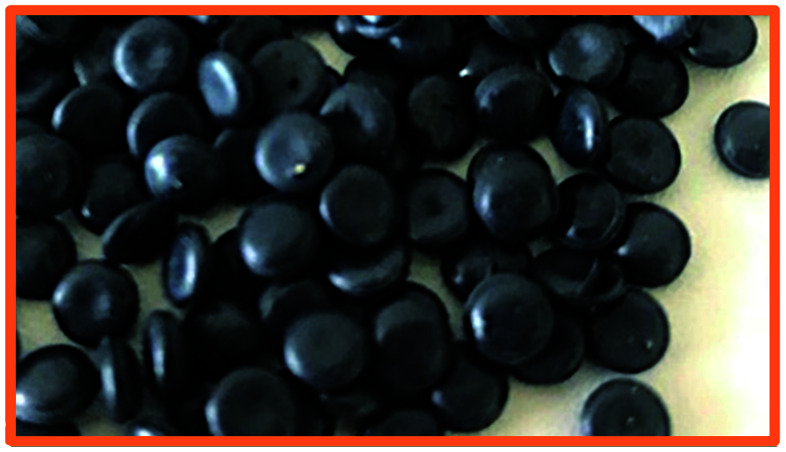	Anthracite spherical granules	Plastics originating from construction foils
PE3	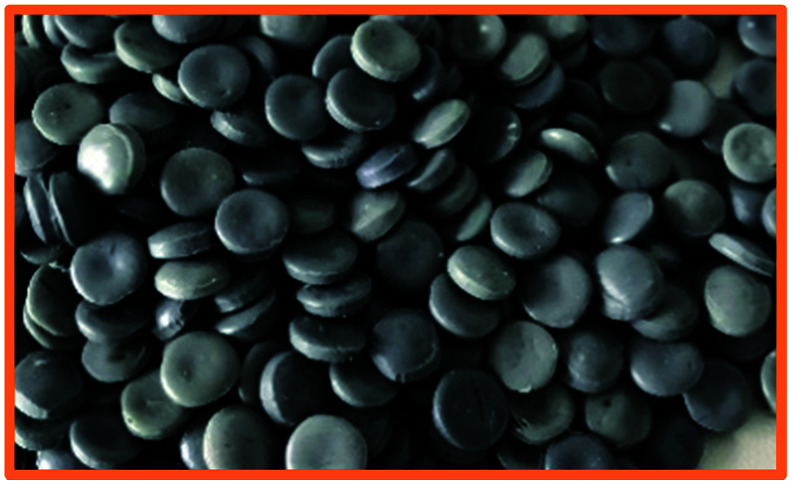	Dark grey spherical pellets	Pre-sorted plastic blends from post-consumer packaging plastic waste
PE4	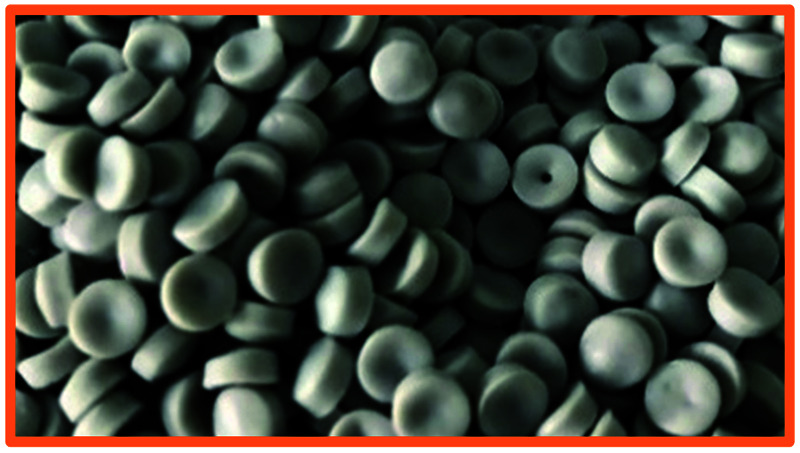	Light grey cylindrical pellets	Multi-phase sorted plastic blends from post-consumer packaging plastic waste
PE5	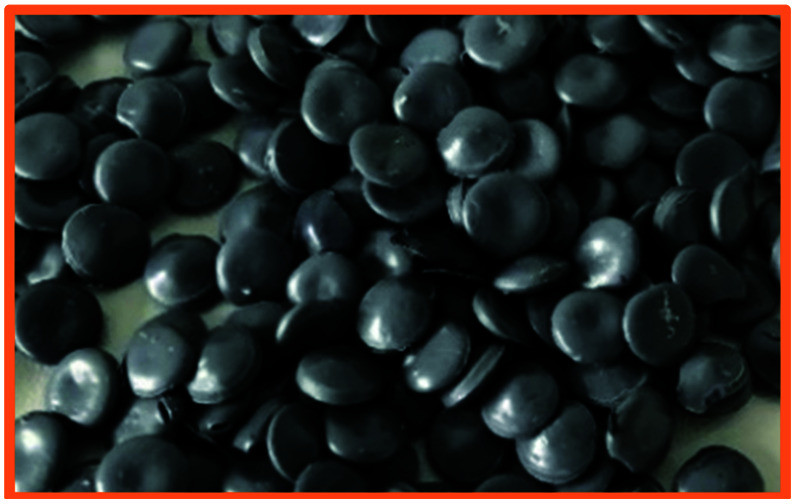	Dark grey spherical pellets	Pre-sorted plastic blends from post-consumer packaging plastic waste
PE6	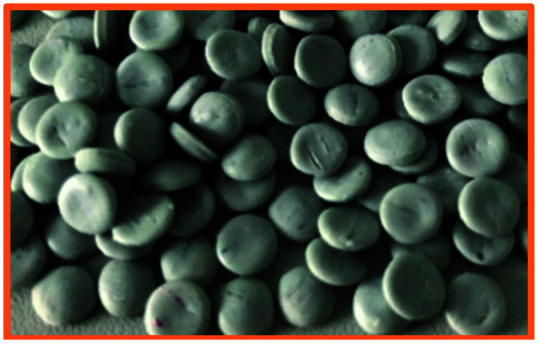	Light grey spherical pellets	Multi-phase sorted plastic blends from post-consumer packaging plastic waste
PP1	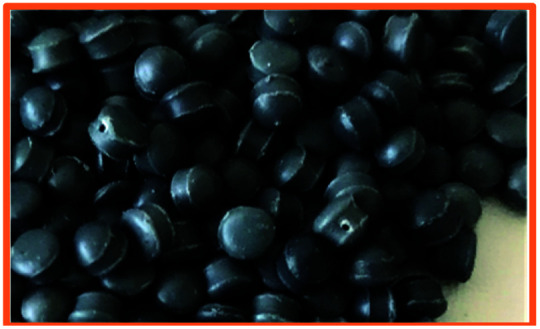	Anthracite cylindrical pellets	Pre-sorted plastic blends of mixed origin (post-industrial, post-commercial, bulky waste, and post-consumer). A selective recycling technology is applied, including degassing and filtration
PP2	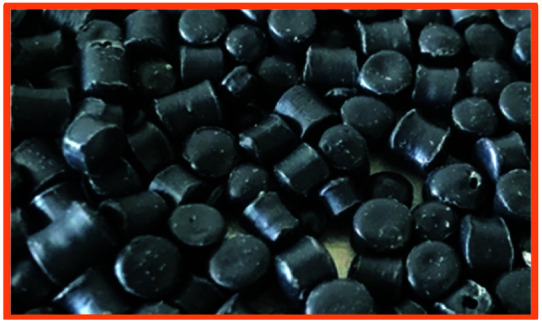	Dark grey cylindrical pellets	Pre-sorted plastic blends of mixed origin (post-industrial, post-commercial, bulky waste, and post-consumer). A selective recycling technology is applied, including degassing and filtration

#### Recycled polymers with further processing

Only HDPE (PE2) was considered, and two main aspects were evaluated:

• The chemical quality of the recycled material in relation to the product's composition (*i.e.* the fraction of recycled plastic to virgin plastic: recycled/virgin (wt%)).

• The degree of the chemical quality deterioration (contamination) as a function of the number of applied extrusion cycles.

To arrange the test sets (samples with different compositions of recyclate to virgin (RE : VI)), five main mixtures were prepared by extruding the following RE : VI mass fractions: 0 : 100 wt%, 25 : 75 wt%, 50 : 50 wt%, 75 : 25 wt%, and 100 : 0 wt%. The extrusion was conducted using HAAKE™ Rheomex CTW 100 OS, a twin-screw extruder from Thermo Fisher Scientific.

The produced filaments were separately shredded to the size of 4 mm × 4 mm. The shredded material of each mixture was separately collected for further testing.

### Analysis of the PAH content in the plastic samples

To prevent cross-contamination, only glassware was used to conduct the extraction, treatment and analysis of samples. In order to analyse the PAH content in the plastic matrix, samples underwent extraction following the method reported by Alassali *et al.*^26^ Randall hot extraction was used as suggested by Geiss *et al.*^20^ The PAH content analysis was conducted on the extract after applying a clean-up and drying steps (as explained below).

#### PAH extraction from the plastic matrix

To extract PAH from the plastic's matrix, Randall hot extraction was conducted using Behr E4 from Behr Labor-Technik GmbH. The extraction was applied on 0.1 to 0.2 g of a sample. 75 mL of toluene was used as the extraction solvent in accordance with literature.^15,20,27^ The first extraction step consisted of 120 min of sample immersion in toluene at 140 °C, followed by a washing step at 170 °C for 60 min. Finally, a solvent recovery step (evaporation step) was applied at 170 °C, until the target volume of ≤20 mL was reached.^26,28^

After the evaporation step, the reaction vessels were cooled down to room temperature. An internal standard solution was added to the solution to calculate the recovery. The extract was then filtered into a 100 mL pear-shaped flask to remove the plastic particles from the extract. The extract's volume was decreased to ∼500 μL by evaporation using a 0.7 bar nitrogen stream at room temperature.

#### Extract clean-up

The clean-up was done by solid phase extraction (SPE), using silica gel disposable extraction columns.

The procedure of SPE consisted of the following steps in the given order:

1. Cleaning of the columns using dried CH_2_Cl_2_/hexane mixture.

2. Conditioning of the columns with dried *n*-hexane.

3. Sample application.

4. Sample washing using 500 μL of *n*-hexane, added to the pear-shaped flask containing the sample for rinsing purposes. After rinsing, the solution was added to the column.

5. Elution applying dried CH_2_Cl_2_/hexane.

6. The eluate was collected in clean test tubes.

7. Eluate concentration to 500 μL using a 0.5 bar of N_2_ stream at room temperature.

#### Analysis

The analysis of the extract was performed using a gas chromatograph HP 6890 coupled with a single ion monitoring mass selective detector HP 5973 from Agilent Technologies.^26^ The used GC-column is HP-5MS, 30 m long, with 0.25 mm internal diameter. After the sample injection, the temperature was set at 60 °C for 1 min, then it was increased at rate of 20 °C min^−1^ until it reached 160 °C. The temperature was further increased, at a rate of 10 °C min^−1^, to 325 °C. A calibration step preceded the analysis. For the calibration, different established concentrations between 10 and 1000 ng mL^−1^ of an external standard containing the 16-US-EPA PAH were analysed, and the respective responses were obtained. The results were then plotted against the concentration in a linear relationship. Finally, the trend-line was added, and the correlation equation and the coefficient of determination were calculated.

## Results and discussion

### PAH in high density polythene (HDPE) recyclates

**Fig. 1:**
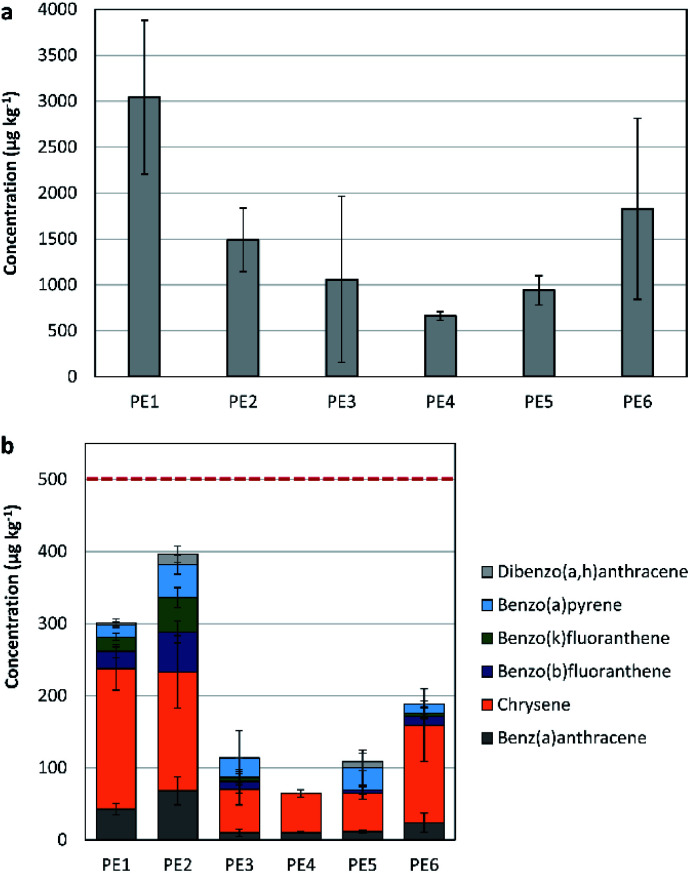
(a) The concentration of the 16-US-EPA PAH in 6 different HDPE recyclate samples. (b) The content of 6 out of 8 REACH priority PAH in HDPE recyclates. The red line is the threshold limit provided by REACH for children articles.

The content of the total 16-US-EPA PAH was studied in 6 different HDPE recyclate samples, to assess their applicability in different product categories (see Fig. 1(a)). The average concentration of the 16-US-EPA PAH in the recyclate samples ranged between 419.7 and 3855.4 μg kg^−1^.

The highest PAH concentration was observed in PE1 (3043 ± 837.7 μg kg^−1^), a sample of a mixed source (post-consumer, post-commercial, and post-industrial waste).

The lowest total PAH concentration in the tested recyclates was obtained in PE4 (662 ± 47.7 μg kg^−1^), a sample originating from post-consumer packaging waste that is light grey in colour. This sample underwent multiple sorting steps (by plastic type and colour) before recycling.

The normality test (Shapiro–Wilk) on the content of the 16 PAH in the different samples passed (*P* = 0.6), while the equal variance test (Brown–Forsythe) failed (*P* < 0.05). Applying ANOVA on ranks showed that there is not a statistically significant difference for the different HDPE recyclate samples (*P* = 0.074).

It was reported by Camacho *et al.*^21^ that the concentration of aromatic hydrocarbons in the recycled resin is approximately five times higher in comparison to virgin PE and PP. This research indicated that the content of the PAH in recyclates is 4 to 20 times higher than that in pristine resins, depending on the materials' source and the efficiency of sorting.

Analysing the individual 16-US-EPA PAH in all recycled samples showed significant variances in the phenanthrene and pyrene contents. Phenanthrene is present in fossil fuels and derived products. Like most PAH, phenanthrene is present in manufactured dyes, plastics, and pesticides.^29^

Hence, its high concentration in the recyclates is attributed to its presence in the plastic waste put for recycling (either as additive or as impurity). Pyrene was found in high concentrations in crude oil and in extender oil.^15^ Hence, its presence is attributed to the production process of plastics.

Among the 16-US-EPA PAH, 6 belong to the REACH's 8 priority PAH list (*i.e.* benzo[*a*]anthracene, chrysene, benzo[*b*]fluoranthene, benzo[*k*]fluoranthene, benzo[*a*]pyrene, and dibenzo[*a*,*h*]anthracene). Both, benzo[*j*]fluoranthene and benzo[*e*]pyrene were therefore not detected. Consequently, when the total concentration of the 6 out of the 8 REACH-priority PAH is slightly below the threshold limits provided by REACH, there is a strong evidence that the sample obtains hazardous properties. PE1 and PE2 had the highest concentrations of the 6 priority PAH, with values of 301 ± 52.3 μg kg^−1^ (*n* = 3) in PE1 and 396 ± 109.1 μg kg^−1^ (*n* = 4) in PE2 (see Fig. 1(b)). The threshold limits for the 8 priority PAH are 500 μg kg^−1^ for children articles, and 1000 μg kg^−1^ for all other consumer products.^20^ Hence, samples PE1 and PE2 might be restricted in their application as recyclates, particularly their application in children articles. Samples PE3, PE4, PE5, and PE6 originate from post-consumer plastic packaging waste, and they showed low level of contamination with the 6 priority PAH (<200 μg kg^−1^).

The concentration of the 6 out of 8-REACH priority PAH for the different samples showed differences in the mean values that are greater than what would be expected by chance; there is a statistically significant difference (*P* = 0.010).

The difference between samples of mixed sources and the ones originating from post-consumer plastic packaging waste was mostly detected in the concentrations of benzo[*a*]anthracene, chrysene, benzo[*b*]fluoranthene, and benzo[*k*]fluoranthene. Benzo[*b*]fluoranthene, and benzo[*k*]fluoranthene are mainly found in crude oil and in extender oil. Chrysene and benzo[*a*]anthracene are found in extender oil, in crude oil, in addition to their presence in carbon black (in lower concentrations).^15^

A review on plastics production and related patents showed that the content of extender oils in plastics is in the range of 2 to 50 wt%. For most of the PAH, only 0 to 5% of the incoming PAH originate from carbon black in comparison to crude oil. It was also reported that the applied production temperature has an influence on the content of the PAH in carbon black, which varies for the different PAH.^15^ Hence, the variance in the different PAH contents in the different samples could be ascribed to the degree of contamination of the source material (by production and/or by use), as well as the temperature applied during recycling, which could have had an effect on the organic additives. Also, a possible source of PAH are the additives added to recyclates (*e.g.* extender oil and/or carbon black).

In May 2019, the German Product Safety Committee (Ausschuss für Produktsicherheit, AfPS) issued new specifications for PAH under the voluntary ‘Tested Safety Mark’ (Geprüfte Sicherheit Mark (GS-Mark)).^30^ There are 3 different categories of products, differing in the repetitiveness and pathway of exposure, as well as in the targeted group (babies, children or adults).

According to the provided limits for each category, samples PE1, PE2, and PE6 did not comply with the specifications for products in category 1 (the concentrations of pyrene, fluoranthene, anthracene, and phenanthrene combined exceeded the limit of 1000 μg kg^−1^). For the rest of categories, all tested samples complied with the defined specifications. This provides that recycled HDPE has to be assessed before being utilised in production to define its applicability for the different products’ categories.

### PAH in polypropylene (PP) recyclates

**Fig. 2:**
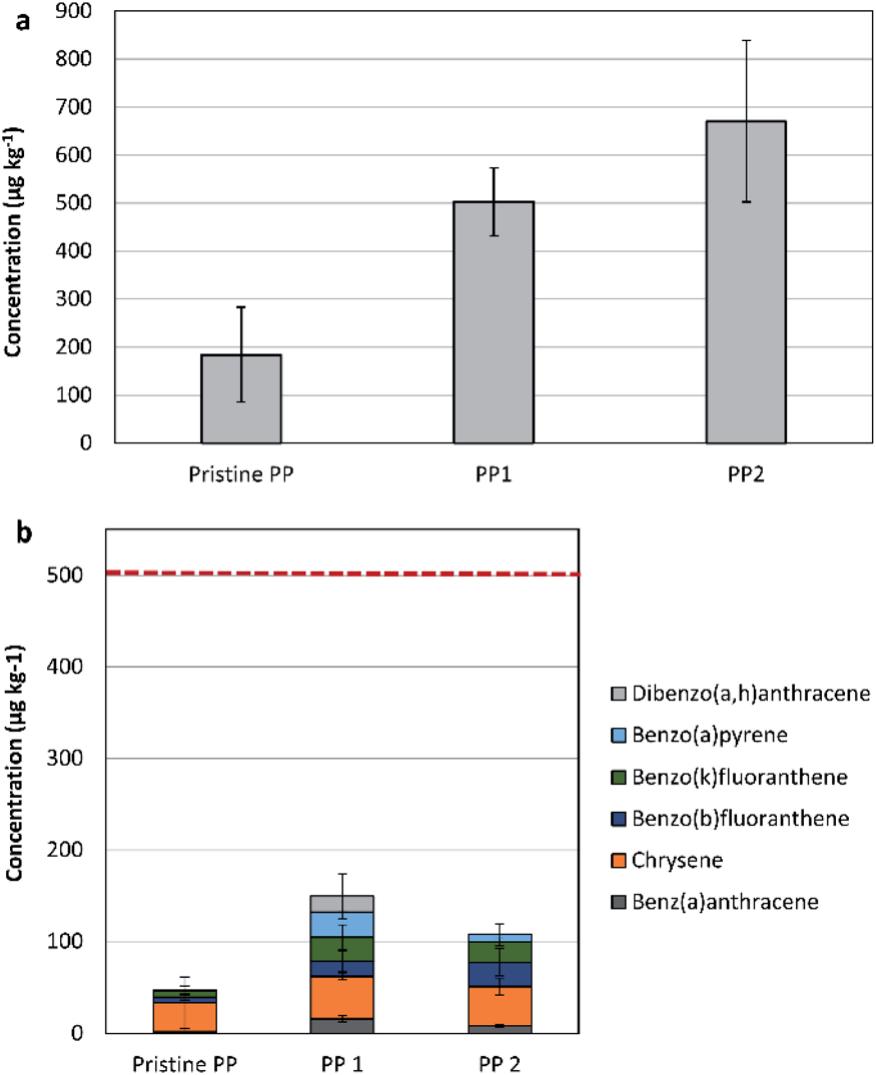
(a) The concentration of the 16-US-EPA PAH in different PP recyclate samples. (b) The content of 6 out of 8 REACH priority PAH in the virgin PP as well as in two PP recyclate samples. The red line is the threshold limit provided by REACH for children articles.

The two tested recycled PP samples are from a mixed source. The concentration of the 16-US-EPA PAH in the purchased PP recyclates showed an increase by 173% for PP1 and by 264% for PP2, in comparison to the pristine PP samples (see Fig. 2(a)). The anthracite sample (PP1) showed lower concentrations of the 16-US-EPA PAH in comparison to the dark grey sample. This is attributed to the extremely selective processing technology applied for recycling, where degassing and very fine filtration were applied.

In comparison to the pristine PP sample, the concentration of phenanthrene was higher by 159% and by 479% in PP1 and PP2, respectively. The concentrations of fluoranthene and pyrene were higher by 153% and 93% in PP1. A similar trend was seen in PP2, fluoranthene and pyrene concentrations were higher by 277% and 209%, respectively, when compared to their concentrations in the pristine PP resin.

By focusing on the 6 out of the 8-REACH priority PAH, the concentrations were in both recyclates below the REACH-indicated threshold limits for both children articles and consumer products. Hence, and due to the low contamination risk, the recycled PP samples indicated suitability for application without restriction (see Fig. 2(b)).

Both PAH compounds, benzo[*a*]pyrene and dibenzo[*a*,*h*]anthracene, were absent in the virgin and pure PP samples. However, benzo[*a*]pyrene was found in PP1 and PP2, while dibenzo[*a*,*h*]anthracene was only found in P1 (anthracite in colour). These products are found in crude oil in higher concentrations in comparison to extender oil. The carbon black was reported to be free of dibenzo[*a*,*h*]anthracene, but containing minor concentrations of benzo[*a*]pyrene (200 to 400 μg kg^−1^).

Accordingly, the greater PAH concentrations in recycled PP sample could be attributed to the original source of material, the processing conditions, and the existing additives (colouring agents and lubricants).^7,31^ Additionally, there is the possibility of environmental contamination, where plastics are expected to absorbed PAH from the surrounding environment.^32^

Measuring the individual concentrations of benzo[*a*]pyrene, benzo[*e*]pyrene, benzo[*a*]anthracene, benzo[*b*]fluoranthene, benzo[*j*]fluoranthene, benzo[*k*]fluoranthene, chrysene, dibenzo[*a*,*h*]anthracene, and benzo[*ghi*]perylene indicated that both recycled PP samples comply with the specifications provided by the German product safety committee (Ausschuss für Produktsicherheit, AfPS) for all products' categories.^30^

According to the obtained results, both recycled PP samples are applicable without limitation since they comply with REACH regulation and with the German product safety committee specifications.

### Assessing the impact of plastic recycling while eliminating the influence of additives

The impact of plastic recycling was simulated in the lab on pristine polymers (LDPE, PP). Up to three extrusion cycles were implemented to augment the influences of heat and stress obtained by recycling. A temperature range of 190 °C to 210 °C was applied, depending on the extrusion cycle. The content of PAH was then compared between the new plastic sample and the one that underwent 3-cycles of extrusion (as the worst case).

**Fig. 3:**
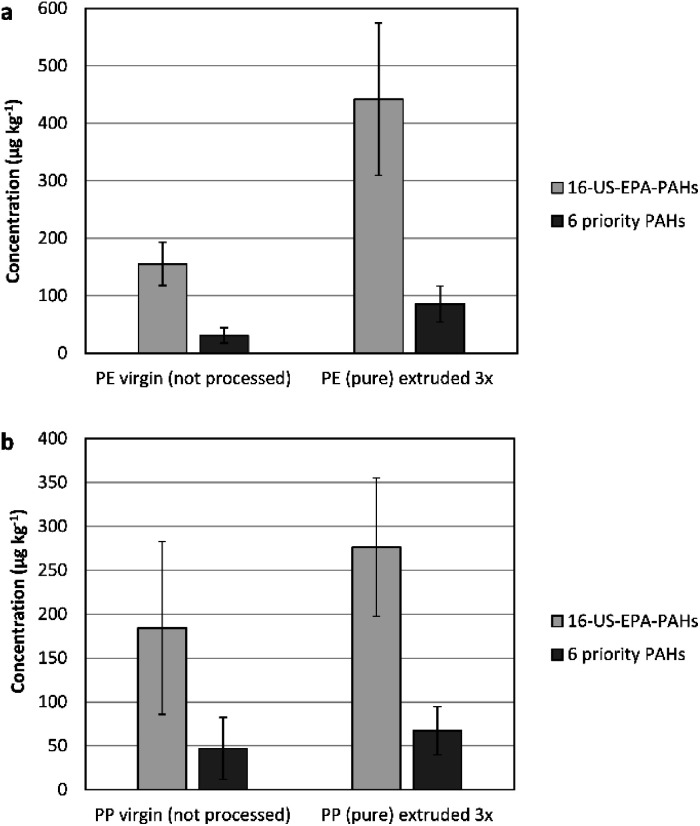
(a) A comparison of the PAH content between pristine PE and 3-cycles extruded pure PE. (b) The PAH content of pristine PP and 3-cycles extruded pure PP.

For PE 3×-extruded sample, the 16-US-EPA PAH concentrations increased by 185% (from 155 ± 37.9 μg kg^−1^ to 442 ± 133 μg kg^−1^). A similar factor of increase was obtained for the 6-priority PAH, showing that there is a general trend of increase (see Fig. 3(a)).

The increase of the concentrations of naphthalene, phenanthrene, fluoranthene, pyrene, and chrysene were most significant in comparison to the rest of analysed PAH (increasing by 200 to 300%).

The same trend was observed for PP. A minor increase in the total concentration of PAH was observed in pristine PP after extrusion. However, the percentage of increase was lower by a factor of 43.6% than that for the LDPE polymer. Even a lesser percentage of increase was obtained for the 6-priority PAH (29.5%) (see Fig. 3(b)).

In general, the temperature applied during extrusion is insufficient to cause complex reactions that could result in the formation of PAH from within the polymers. Since plastics are in particular sensitive to absorbing low molecular weight compounds due to their permeable nature, the increase in the PAH content could be explained as an accumulation from the surrounding environment.^21^ The difference in the PAH degree of increase between HDPE and PP is a proof on that, where HDPE is reported to have higher sorption capacity to PAH in comparison to PP.^33^

Overall, despite the increase in the PAH content in pristine HDPE and PP after extrusion, the PAH content was tangibly lower than the threshold limits for both, children articles and consumer products.

### Assessing the impact of further recycling of plastics containing additives on the PAH content

**Fig. 4:**
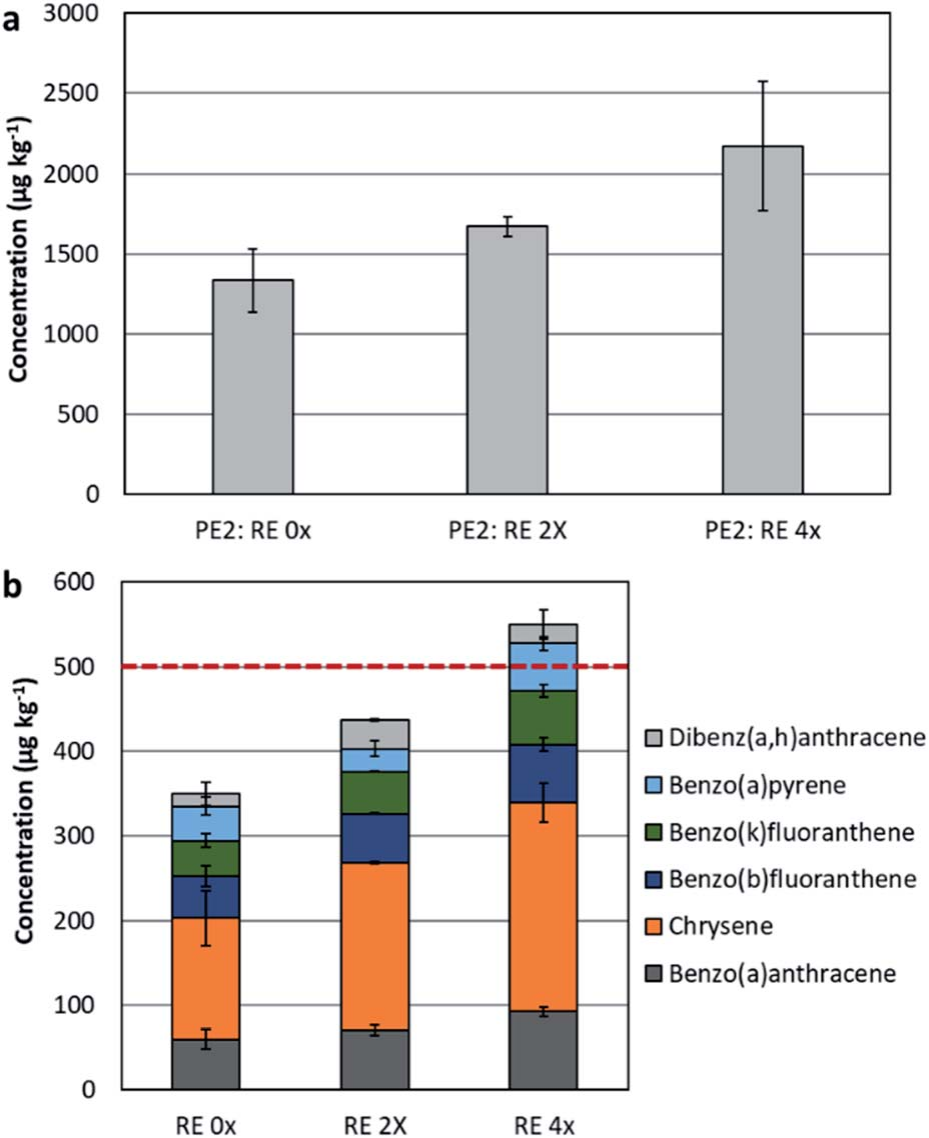
(a) The concentration of the 16-US-EPA PAH in PE2 recyclates, before (RE 0×) and after undergoing further extrusion (2× and 4×). (b) The concentration of the 6 out of 8 REACH priority PAH in the respective samples, the red line is the threshold limit provided by REACH to the 8-priority PAH in children articles.

In this section, the impact of extrusion was assessed on recyclate PE2. Recyclates usually contain additives, fillers, lubricants, and colouring agents, either originating from the source material put for recycling or by additives put at the recycling stage to obtain the needed quality. Therefore, the effect of recycling of plastics containing additives could be here assessed. The settings of the mechanical recycling process were simulated to test the influence of heating on PAH formation. The extrusion was conducted up to four consecutive cycles, and only samples underwent 2 extrusion cycles (PE2: extruded 2×) and 4 extrusion cycles (PE2: extruded 4×) were tested. Generally, the sample that was extruded twice had slightly higher PAH concentrations in comparison to the original sample. Yet, when extrusion was applied up to four times, the increase was more significant and with higher standard deviations, in other words less uniformity in the PAH content (see Fig. 4(a)).

Extrusion resulted in a significant rise in the content of: fluorene, phenanthrene, fluoranthene, pyrene, benzo[*a*]anthracene, and chrysene. Applying the limits provided by the Regulation EU 1272/2013 amending REACH Annex XVII, where 6 out the 8 indicators were considered (*i.e.* benzo[*a*]pyrene, benzo[*a*]anthracene, chrysene, benzo[*b*]fluoranthene, benzo[*k*]fluoranthene, dibenzo[*a*,*h*]anthracene), the 2×-extruded sample and the 4×-extruded samples had a sum of 436 ± 13.0 μg kg^−1^ and 550 ± 29.5 μg kg^−1^, respectively (original sample had a concentration of 349 ± 67.5 μg kg^−1^).

This concludes that these samples are not allowed to be utilised in producing children articles (see Fig. 4(b)), yet applicable in consumer products.

According to the German product safety committee (Ausschuss für Produktsicherheit, AfPS), the concentration of chrysene in the 2×-extruded PE2 and the 4×-extruded PE2 was exceeding the threshold limit for products in category 1 (limit value is 200 μg kg^−1^). A similar observation was seen for the sum of concentrations of phenanthrene, anthracene, fluoranthene, and pyrene, which was as well exceeding the limit (1000 μg kg^−1^) for products in category 1.

### The impact of mixing recyclates with new polymers (dilution) on the PAH content

Generally, recyclates dilution with virgin and pure polymers resulted in a decrease in the total concentration of the 16-US-EPA PAH. It was observed that the decrease was 16% for the 50 wt%-RE sample and improved by almost the double (decrease by 30%) for the 25 wt%-RE.

Phenanthrene was lost in both diluted samples (*i.e.* 50%-RE and 25%-RE). This could be attributed to the low melting point of phenanthrene (99 °C)^34^ in comparison to the temperature applied during extrusion. During PE2 extrusion, phenanthrene can experience melting and a consequent vaporisation. Additionally, the dilution of the material resulted in further decrease in its concentration to be undetectable.

On the other hand, a substantial increase in the concentration of pyrene was observed in both 50 wt%-RE and 25 wt%-RE samples, in comparison to the 100 wt%-RE sample (243 ± 56.1 μg kg^−1^ in 100 wt%-RE, 571 ± 152 μg kg^−1^ in 50 wt%-RE, and 382 ± 59.3 μg kg^−1^ in 25 wt%-RE). The general increase in the pyrene's concentration could be due to the inhomogeneous mixing of additives in the extruder and the successive heating, which doesn't apply to the 100 wt%-RE sample (the original material).

## Conclusions

This research studied the degree of recyclates contamination with PAH in relation to the mechanical recycling process. First, the PAH content was tested in polyolefin recyclates to assess their applicability in the different products' categories. Second, the impact of material mechanical processing on the PAH content was assessed by analysing pure polymers before and after undergoing a simulated recycling process. Testing pure polymers was important to exclude possible contamination with PAH additives.

Generally, recyclates originating from post-consumer plastic waste had lower concentrations of the 16-US-EPA PAH (922 ± 420.8 μg kg^−1^), in comparison to the recyclates obtained from mixed origins (2155 ± 991.9 μg kg^−1^), *r* = −0.35, *p* > 0.05.

The degree of recyclates contamination with PAH was within the REACH limits for consumer products for all tested samples. However, polythene recyclates originating from post-commercial waste did not comply with the REACH limits for children articles (0.5 mg kg^−1^). Hence, the quality of recyclates has to be assessed before being considered for application in children articles.

As per the results, it is concluded that the recycling process could contribute to the formation and accumulation of PAH in plastics. Yet the increase is insignificant in pure plastics, indicating that the previous presence of PAH additives is the limiting factor. Hence, the design and source of plastic waste define the quality of the derived recyclates. This indicates the need of having stricter products' specification in terms of additives quality and quantity in the original products. Furthermore, in addition to the plastic waste separate collection, enhanced sorting can provide an improved quality of recyclates.

Additionally, the results recommend having a controlled environment when recycling is applied to minimise the possibility of PAH accumulation from the surrounding environment.

All in all, the plastic waste stream is very complex and heterogeneous. Therefore, the number of analysed recyclate samples in this study (6 for HDPE and 2 for PP) is not enough to provide conclusions of high certainty. Consequently, it is recommended to extend the analysis by including larger sets of samples of diverse sources and by considering further contaminants of concern.

## Conflicts of interest

There are no conflicts to declare.

## Supplementary Material
